# The Addition of Sirolimus to GVHD Prophylaxis After Allogeneic Hematopoietic Stem Cell Transplantation: A Meta-Analysis of Efficacy and Safety

**DOI:** 10.3389/fonc.2021.683263

**Published:** 2021-09-09

**Authors:** Xiaoli Chen, Hengrui Sun, Kaniel Cassady, Shijie Yang, Ting Chen, Li Wang, Hongju Yan, Xi Zhang, Yimei Feng

**Affiliations:** ^1^Medical Center of Hematology, The Xinqiao Hospital of Third Military Medical University, Chongqing, China; ^2^Irell and Manella Graduate School of Biological Sciences of City of Hope, Duarte, CA, United States

**Keywords:** sirolimus, GVHD, HSCT, prophylaxis, TMA

## Abstract

**Objective:**

The objective of this study was to evaluate the safety and efficacy of sirolimus (SRL) in the prevention of graft-*versus*-host disease (GVHD) in recipients following allogeneic hematopoietic stem cell transplantation (allo-HSCT).

**Methods:**

Randomized controlled trials (RCTs) evaluating the safety and efficacy of SRL-based prophylaxis regimens in patients receiving allo-HSCT were obtained from PubMed, Embase, and the Cochrane database. Following specific inclusion and exclusion criteria, studies were selected and screened by two independent reviewers who subsequently extracted the study data. The Cochrane risk bias evaluation tool was used for quality evaluation, and RevMan 5.3 software was used for statistical analysis comparing the effects of SRL-based and non–SRL-based regimens on acute GVHD, chronic GVHD, overall survival (OS), relapse rate, non-relapse mortality (NRM), thrombotic microangiopathy (TMA), and veno-occlusive disease (VOD).

**Results:**

Seven studies were included in this meta-analysis, with a total sample size of 1,673 cases, including 778 cases of patients receiving SRL-based regimens and 895 cases in which patients received non-SRL-based regimens. Our data revealed that SRL containing prophylaxis can effectively reduce the incidence of grade II–IV acute GVHD (RR = 0.75, 95% CI: 0.68∼0.82, *p* < 0.0001). SRL-based prophylaxis was not associated with an improvement of grade III–IV acute GVHD (RR = 0.78, 95% CI: 0.59∼1.03, *p* = 0.08), chronic GVHD (*p* = 0.89), OS (*p* = 0.98), and relapse rate (*p* = 0.16). Despite its immunosuppressant effects, SRL-based regimens did not increase bacterial (*p* = 0.68), fungal (*p* = 0.70), or CMV (*p* = 0.10) infections. However, patients receiving SRL-based regimens had increased TMA (*p* < 0.00001) and VOD (*p* < 0.00001).

**Conclusions:**

This meta-analysis indicates that addition of sirolimus is an effective alternative prophylaxis strategy for II–IV aGVHD but may cause endothelial cell injury and result in secondary TMA or VOD events.

## Introduction

Allogeneic hematopoietic stem cell transplantation (allo-HSCT) is currently one of the most effective means to cure hematological malignancies. However, high incidence of graft *versus* host disease (GVHD) after transplantation results in high non-recurrence of transplantation-related death. The incidence of acute GVHD (aGVHD) is about 40%–75% and is an important factor affecting the overall efficacy of HSCT (1). In addition, chronic GVHD (cGVHD) has become the main cause of late non-relapse mortality (NRM) after HSCT, which also seriously affects the efficacy of transplantation and the quality of patient life. Currently, GVHD prophylaxis regimens among transplant centers are not uniform and mainly include calcineurin inhibitor (CNI), sirolimus (SRL), and posttransplantation cyclophosphamide (PT-Cy)-based regimens.

Our study focuses on the role of SRL in GVHD. SRL is an mTOR inhibitor, possessing antifungal, immunosuppressive, and antitumor properties (2). SRL can inhibit the proliferation and activation of T cells, reduce the release of pro-inflammatory cytokines, and modulate CD4^+^CD25^+^ regulatory T (Treg) cells, making it a widely used therapeutic candidate in benign and malignant hematological diseases (3). Accumulating evidence suggests that SRL may play a role in the prevention and treatment of GVHD after HSCT (4). However, some studies have reported that SRL-based regimens did not decrease the incidence of aGVHD (5, 6). Moreover, some have reported that SRL-based prophylaxis was associated with high incidence of thrombotic microangiopathy (TMA) (7). On the other hand, another group reported that the combination of tacrolimus (TAC)/SRL did not pose a higher risk of TMA (8). Therefore, to better understand the efficacy and safety of SRL-based regimens and their impact on GVHD, we performed a meta-analysis of SRL-based GVHD prophylaxis in patients after allo-HSCT.

## Materials and Methods

### Search Strategy

A literature search was conducted to identify randomized controlled trials (RCTs) evaluating the efficacy of SRL-based prophylaxis in patients after allo-HSCT. The search was conducted through August 2020 in PubMed, Cochrane Library, Embase, and Web of Science. The search terms included “sirolimus,” “rapamycin,” “graft *versus* host disease” and “GVHD.” The search language was restricted to English.

### Inclusion and Exclusion Criteria

Inclusion criteria: the RCT study must include an SRL-based group and a non-SRL-based prophylaxis group. RCTs included patients with hematological malignancies that have received allo-HSCT. The meta-analysis did not exclude studies or patients based on age, gender, source of donor, and level of radiotherapy and chemotherapy before transplantation. Primary outcomes included the incidence of aGVHD and cGVHD. The secondary outcomes included TMA, VOD, and overall survival (OS).

Exclusion criteria are non-RCTs, such as retrospective studies, conference articles, animal experiments, and review articles.

### Data Extraction

All data, including the first author of the studies, published year, country of origin, period of enrollment, sample size, median follow-up duration, SRL-based regimens, non-SRL-based regimens, and trial outcomes, were extracted by two independent researchers. Any discrepancies were resolved by discussion and/or consultations with a third independent researcher.

### Methodologic Quality Evaluation

#### Statistical Analysis

The risk ratio (RR) and 95% confidence interval (CI) were used to analyze extracted dichotomous outcomes. Heterogeneity was assessed using the *I^2^* statistic. An *I^2^* value of greater than 50% and a p value less than 0.10 indicated significant heterogeneity (9). Sensitivity and subgroup analyses were performed to identify and reduce heterogeneity. Meta-analyses were conducted using random effects, regardless of the existence or non-existence of heterogeneity.

## Results

### Study Selection and Characteristics

**Figure 1:**
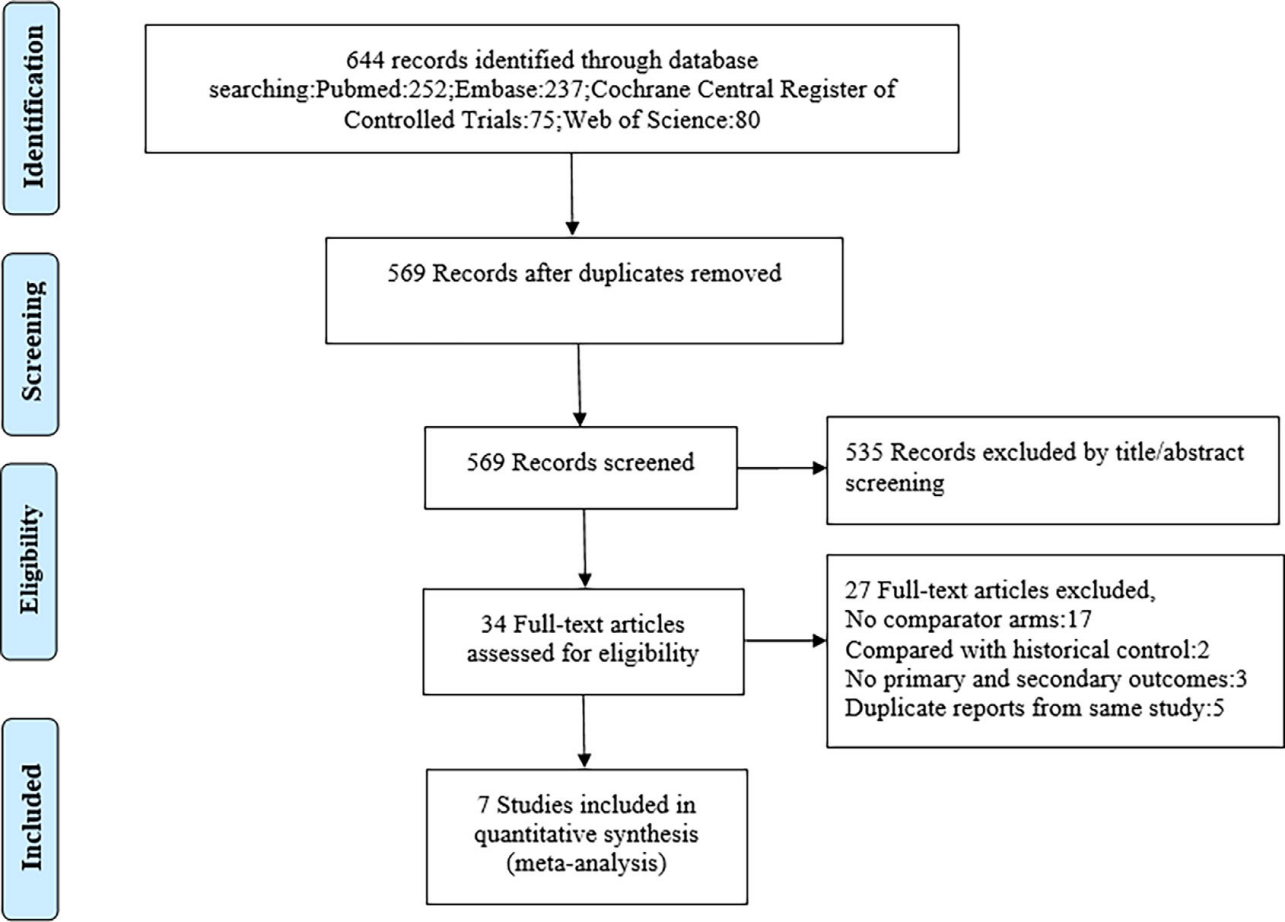
Flow diagram of this study.

In total, 644 potentially relevant records were identified in database records using the selected search terms **(** **Figure 1** **)**. After a thorough screening of the remaining 569 titles and abstracts, 535 non-relevant studies were excluded. The full texts of the remaining 34 studies were assessed, leading to the elimination of 27 studies that did not meet the eligibility criteria. Subsequently, the remaining seven studies were included in our meta-analysis. Characteristics of the studies included in this analysis are listed in **Tables 1**, **2**. Totally, seven studies were included ranging from 74 to 707 patients. In five of these studies, the prophylactic regimen was CNI + methotrexate (MTX) *vs*. CNI + MTX + SRL (10–14). In two studies, CNI + mycophenolate mofetil (MMF) was compared with CNI + MMF + SRL (15, 16) for GVHD prophylaxis.

**Table 1:** Characteristics of the included RCTs, comparing the SRL-based and non–SRL-based groups.

First author and year	Disease	Age (SRL group)	Sample size SRL/non-SRL	Donors	Conditioning regimen
Armand 2016 (10)	Lymphoma, except Burkitt lymphoma.	57 (23–70)	66/73	HLA-matched related donors or MUD.	RIC regimen
Pulsipher 2014 (11)	High-risk ALL in CR	NA (1–21)	73/70	HLA-matched siblings, HLA-matched related or unrelated donors, or single cord blood unit with 4–6/6 matched.	Myeloablative regimen (TBI followed by thiotepa or etoposide and CY)
Cutler 2014 (12)	Acute leukemia in remission, MDS, or CML.	45 (19–59)	151/153	HLA-matched sibling.	Myeloablative regimen (TBI in combination with either CY or etoposide)
Khimani 2017 (13)	AML, MDS, CML, ALL, CLL, sAA, MM, and lymphoma	52 (19–74)	293/414	HLA-matched sibling HLA-matched or mismatched unrelated donors	Standard myeloablative Escalated dose busulfan Non-myeloablative Reduced toxicity
Pidala 2015 (14)	AML, MDS, CML, ALL, CLL, sAA, MM, and lymphoma	49 (25–68)	37/37	Only 8/8 or more HLA-matched sibling or unrelated donors	Bu/pent, Flu/Mel, Flu/Mel
Sandmaier 2019 (15)	Advanced hematological malignancies.	63 (58–68)	90/77	At least 9/10 HLA-matched unrelated donors	Fludarabine+TBI
Kornblit 2014 (16)	AML, ALL, MDS, CML, CLL, MM, and lymphoma	61 (15–76)	68/71	HLA-matched or mismatched unrelated donors	Nonmyeloablative regimen (fludarabine and 2 Gy TBI)

SRL, sirolimus; ALL, acute lymphoblastic leukemia; AML, acute myeloid leukemia; CLL, chronic lymphocytic leukemia; CML, chronic myelogenous leukemia; CR, complete remission; MDS, myelodysplastic syndrome; SAA, severe aplastic anemia; MM, multiple myeloma; HLA, human leukocyte antigen; MUD, matched unrelated donor; URD, unrelated donor; TBI, total body irradiation; CY, cyclophosphamide; RIC, reduced-intensity conditioning; Bu, busulfan; ATG, anti-thymocyte globulin; Flu, fludarabine; Mel, melphalan; NMA, non-myeloablative; HCT, allogeneic hematopoietic cell transplantation.

**Table 2:** Characteristics of the included RCTs, comparing the SRL-based and non–SRL-based groups.

First author and year	SRL usage and dosage	Grouping scheme	SRL administration time	Follow-up time (months)	SRL-based benefit outcomes (*p* < 0.05)
Armand 2016 (10)	12 mg orally on day -3, then 4 mg daily to 360 days, with 5~12 ng/ml	TAC+MTX *vs*. TAC+MTX+SRL	-3 days~+360 days	22 (NA)	II–IV aGVHD, yes; III–IV aGVHD, no; cGVHD, no; relapse, no; TMA, no; PFS, no; OS, no
Pulsipher 2014 (11)	4 mg/m^2^ on day 0, maintaining for 6 months, with 3~12 ng/ml level, followed by a 1-month taper	TAC+MTX *vs*. TAC+MTX+SRL	0 days~+180 days	26 (23~38)	II–IV aGVHD, yes; III–IV aGVHD, no; cGVHD, no; relapse, no; TMA, no; VOD, no; OS, no; NRM, no
Cutler 2014 (12)	Started on day -3 with 12 mg, followed by a daily dose of 4 mg, maintaining a 3~12-ng/ml level	TAC+MTX *vs*. TAC+SRL	-3 days~+100 days	24 (NA)	II–IV aGVHD, no; III–IV aGVHD, yes; cGVHD, no; relapse, no; TMA, no; VOD, no; OS, no; NRM, no
Khimani 2017 (13)	9 mg oral loading dose on day -1, kept at 5–14 ng/ml concentration, and continued for at least 1 year	TAC+MTX *vs*. TAC+SRL	-1 day~+365 days	23.7(11.1-73.1)	II–IV aGVHD, yes; III–IV aGVHD, no; cGVHD, no; relapse, no; VOD, no; OS, yes; NRM, no
Pidala 2015 (14)	Started on day -1 with 9 mg, maintaining 5~14 ng/ml level, continued for at least 1 year	TAC+MTX *vs*. TAC+SRL	-1 day~+365 days	41(27~60)	II–IV aGVHD, yes; cGVHD, yes; relapse, yes; TMA, no; VOD, no; OS, no; NRM, no.
Sandmaier 2019 (15)	Started on day -3 at 2 mg/day, maintaining 3~12 ng/ml to day 150, and tapered off by day 180	CsA+MMF *vs*. CsA+MMF+SRL	-3 days~+180 days	48 (31–60)	II–IV aGVHD, yes; III–IV aGVHD, yes; cGVHD, no; relapse, no; OS, yes; PFS, no; NRM, yes
Kornblit 2014 (16)	Started on day -3 at 2 mg, maintaining 3–12 ng/ml. Stopped on day 80 without a taper	TAC+MMF *vs*. TAC+MMF+SRL	-3 days~+80 days	59 (6–101)	II–IV aGVHD, yes; III–IV aGVHD, no; cGVHD, no; relapse, no; EFS, no; OS, no; PFS, no

TAC, tacrolimus; MTX, methotrexate; CSA, cyclosporine; MMF, mycophenolate mofetil; aGVHD, acute graft-versus-host disease; OS, overall survival; PFS, progression-free survival; NRM, non-relapse mortality; TMA, thrombotic microangiopathy; VOD, veno-occlusive disease.

### Risk-of-Bias Assessment

**Figure 2:**
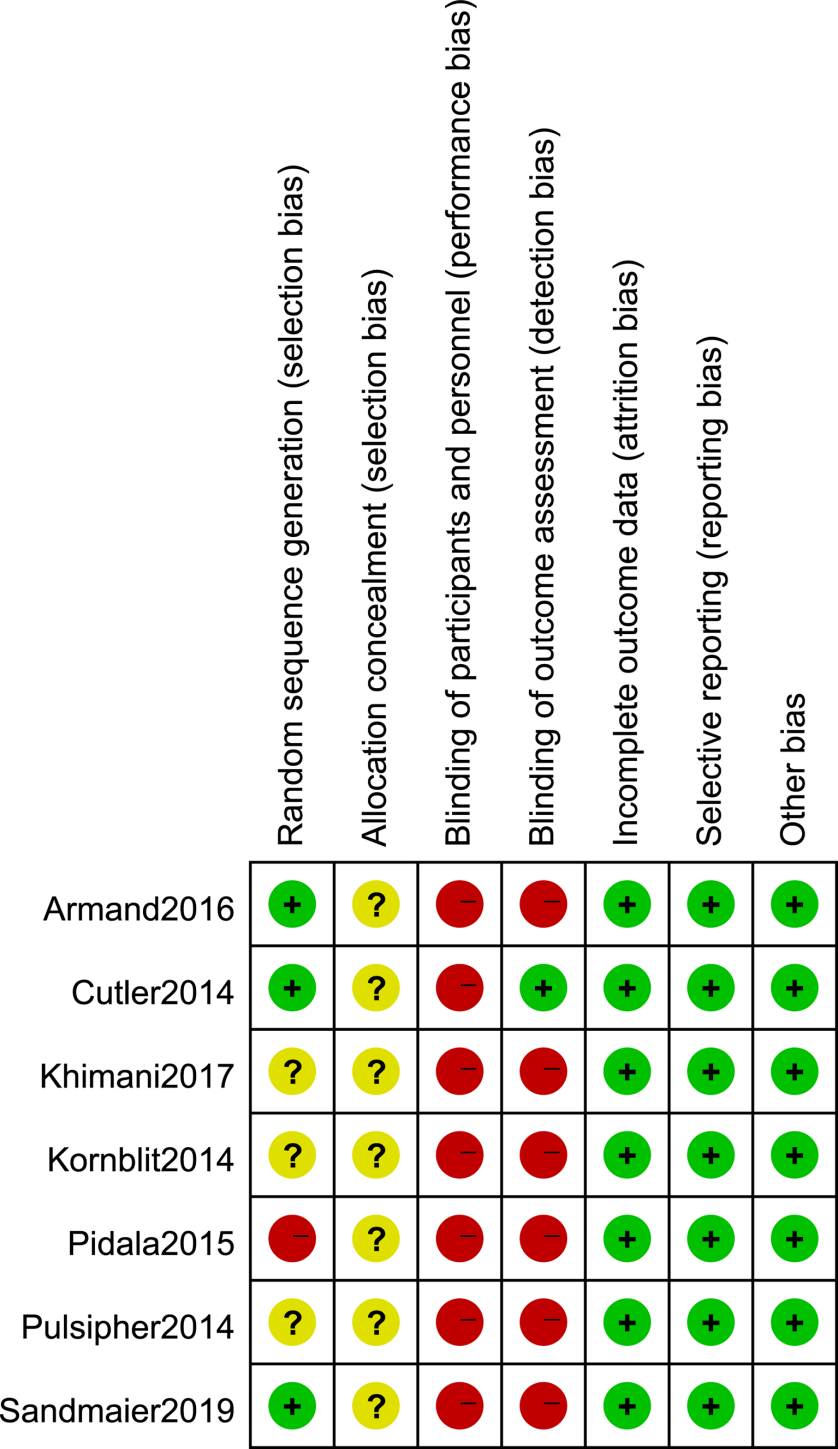
Risk of bias of included randomized controlled trials.

The quality evaluation of the included studies was performed according to the Cochrane handbook. The risk-of-bias assessment was performed to address six aspects: random sequence generation, allocation concealment, blinding of participants and personnel, blinding of outcome assessment, incomplete outcome data, and selective reporting. In the selection bias assessment, three papers described the use of computer-generated random sequences, one paper randomized according to age and donor type, and the other three papers did not mention their grouping method. Seven studies did not mention their method for allocation concealment, while one mentioned the method of blinding data extraction. Seven studies were all low risk on other bias assessment **(** **Figure 2** **)**.

### Study Outcome Analysis

#### Primary Outcomes: GVHD, Including aGVHD and cGVHD

**Figure 3:**
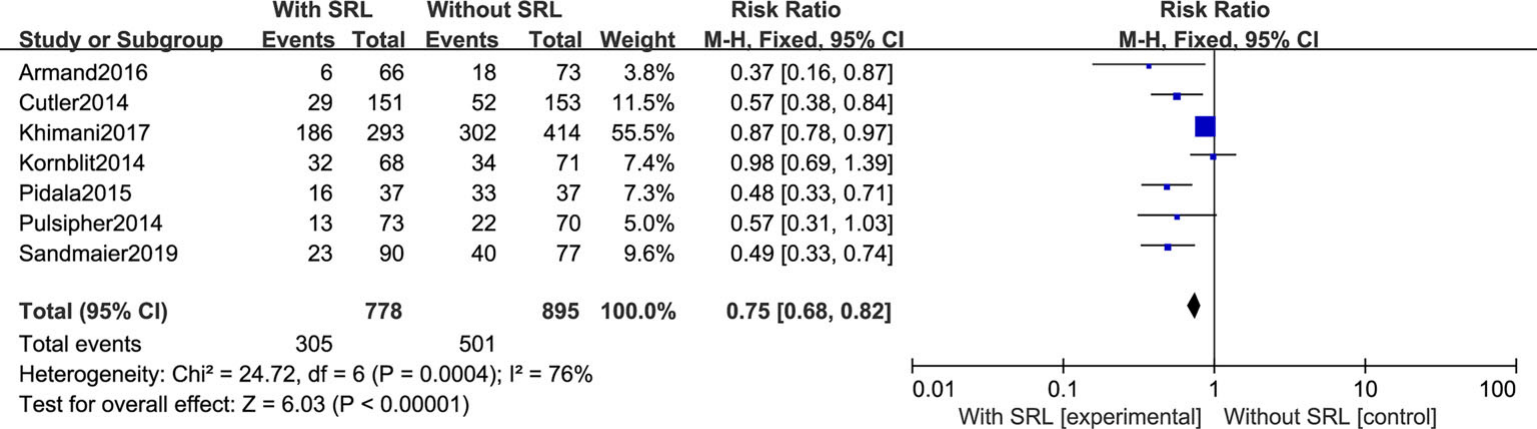
The effect of addition of SRL to prophylaxis on Grade II to IV aGVHD.

**Figure 4:**
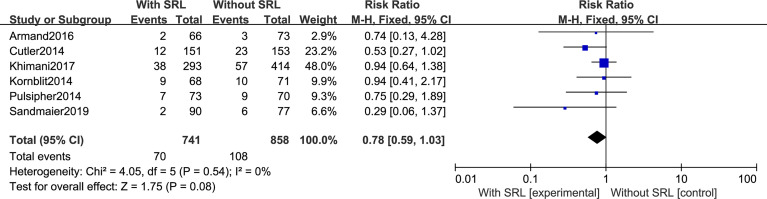
The effect of addition of SRL to prophylaxis on Grade III to IV aGVHD.

All seven studies reported the incidence of Grade II–IV aGVHD. Interstudy heterogeneity was observed to be significant, and the random-effect model was used (*I^2^ = *76%, *p* < 0.0001). By Stata analysis, Begg’s test showed that *p* = 0.548 > 0.5, which suggests that there is no publication bias. Based on the results of modeling, SRL-based regimens significantly decreased the incidence of Grade II–IV aGVHD (RR, 0.75; 95% CI, 0.68–0.82; **Figure 3**). Interestingly, six studies reported the incidence of Grade III–IV aGVHD and the results did not support that addition of SRL can also reduce Grade III–IV aGVHD (RR, 0.78; 95% CI, 0.59–1.03, *p* = 0.08; **Figure 4**).

**Figure 5:**
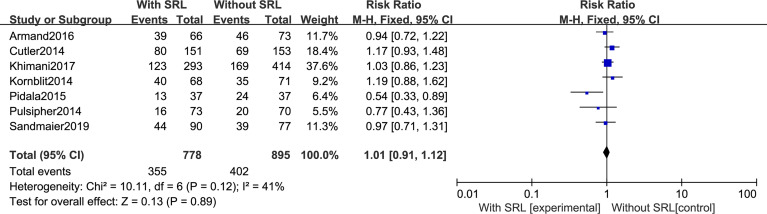
The effect of addition of SRL to prophylaxis on chronic GVHD.

Additionally, SRL-based prophylaxis also cannot reduce the incidence of cGVHD. All seven studies reported that the incidence of cGVHD was not impacted by SRL-based prophylaxis regimens. There was no statistical difference in cGVHD prevention between SRL-based and non-SRL groups (RR, 1.01; 95% CI, 0.91-1.12, *P*=0.89; **Figure 5**).

#### Secondary Outcomes: TMA, VOD, and OS

**Figure 6:**
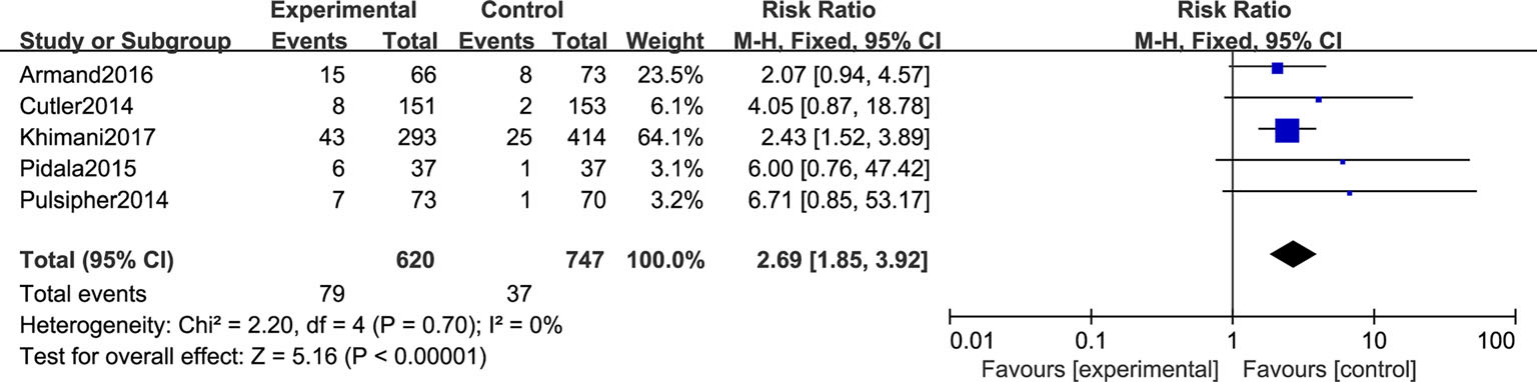
The effect of SRL-based and non-SRL-based prophylaxis on TMA.

**Figure 7:**
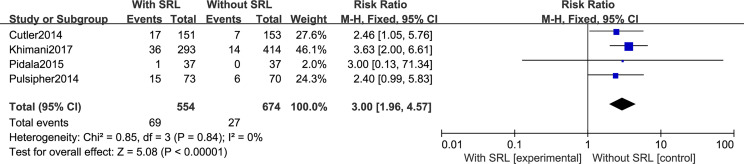
Impact of SRL-based prophylaxis *versus* non-SRL-based prophylaxis on VOD.

Regarding TMA, five studies included in this meta-analysis analyzed the risk of developing TMA. Pooled analysis suggested that all the SRL-based regimens led to an increased TMA incidence (RR, 2.69; 95% CI, 1.85–3.92, *p* < 0.00001; **Figure 6**). Regarding VOD risk, four studies mentioned that SRL-based prophylaxis increased the incidence of VOD, and only one reported that there was no VOD on either arm. Pooled analysis also hinted that SRL-based regimens increase VOD risk (RR, 3.00; 95% CI, 1.96–4.57, *p* < 0.00001; **Figure 7**). It is hypothesized that rapamycin can cause endothelial cell damage through macrophage activation (17) and calcium overload (18).

**Figure 8:**
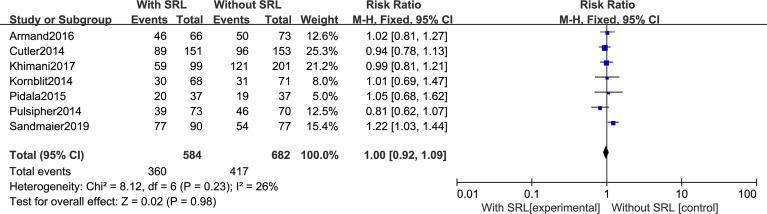
Impact of SRL-based prophylaxis *versus* non-SRL-based prophylaxis on OS.

Our analysis includes seven RCTs to analyze OS. The pooled meta-analysis showed that there are no differences in OS (RR, 1.00; 95% CI, 0.92–1.09, *p* = 0.98; **Figure 8**). Moreover, SRL-based interventions had no statistical effect on PFS, relapse, NRM, bacterial and fungal infection, and CMV reactivation (**Supplementary File 1** **)**.

### Publication Bias Assessment and Sensitivity Analysis

Stata 15.0 software was used to test the publication bias. The results showed that there was no significant publication bias (*p* > 0.05). The heterogeneity of two indexes (II–IV aGVHD and bacterial infection) displayed that the *I^2^* value was greater than 50%. We used sensitivity analysis with Stata software to determine the stability of the results. The sensitivity analysis showed that the research results were stable and reliable **(** **Supplementary File 2** **)**.

## Discussion

Effective prophylaxis of GVHD is the key to successful allo-HSCT. Sirolimus, in combination with a calcineurin inhibitor or post-transplantation cyclophosphamide, is a common regimen for GVHD prophylaxis. This meta-analysis specifically focuses on sirolimus-based GVHD prophylaxis, but there are indeed many other prophylaxis regimens, each with their own advantages and disadvantages depending on the transplant setting and GVHD risk factors.

In this study, we systematically evaluated the effect of SRL on GVHD as a prophylactic drug and included seven RCTs. Statistical results show that SRL prophylaxis regimens can significantly reduce the incidence of Grade II–IV aGVHD. There was no significant difference between the SRL group and the non-SRL group in reducing the incidence of III–IV aGVHD and cGVHD, which indicated that the sirolimus-containing regimen may not prevent all forms of GVHD. This suggests that additional steps may be necessary to prevent cGVHD in the clinic in patients treated with SRL-based regimens.

Meta-analysis showed that the prophylactic regimen containing SRL could reduce acute GVHD but had no effect on OS and PFS. It is known that factors affecting OS and PFS in patients with HSCT are complex, and SRL prophylaxis is not the only factor. Khimani et al. (13) divided the patients into four subgroups according to the hematopoietic cell transplantation–comorbidity index (HCT-CI: creatinine, ejection fraction, FEV1, aspartate aminotransferase, ALT, and bilirubin) (19). Patients with HCT-CI ≥ 4 had significantly worse OS with MTX/TAC than the SRL/TAC group. Thus, it is possible that SRL may improve outcomes in high-risk populations. In addition, SRL combined with post-transplant cyclophosphamide can also provide better survival outcomes in patients after allo-HSCT (20–23). Additional RCTs are required to carefully assess the role of SRL in transplant patient outcomes.

TMA and VOD are both thrombotic diseases that commonly develop post-transplant, and, at present, the pathogenesis of these diseases is unknown. It has been reported that the occurrence of TMA and VOD is related to different regimens, aGVHD, bacterial or fungal infection, HLA mismatch, and combination of drugs (tacrolimus and sirolimus), which caused endothelial injury, thrombosis, and microcirculatory fibrin deposition. Meta-analysis showed that the SRL-based prophylaxes increased the risk of TMA and VOD, suggesting that administration of SRL should be stopped in time when a toxic reaction arises caused by thrombogenesis. However, it should be noted that the occurrence of TMA is not necessarily caused by SRL. Pidala et al. (14) reported that the occurrence of TMA in their study was related to use of tacrolimus, which indicates that TMA may also be a result of the combined effect of SRL and TAC. SRL-induced TMA may be attributable to enhanced platelet activation and aggregation, leading to endothelial damage. Another theory involves the pharmacokinetic interaction between sirolimus and calcineurin inhibitors, which may potentially lead to increased serum and kidney levels of these agents (24). TMA is a multifactorial complication, which may be caused by pathogenic microorganism infection (CMV, BK virus, etc.), calcineurin inhibitor and mTOR inhibitor, GVHD, cytokines (IL-6, INF-a, etc), and neutrophil extracellular traps (NET). TMA might primarily be an effect of high-dose Bu treatment, especially in combination with tacrolimus-based GVHD prophylaxis (25).

The addition of SRL on the basis of the prevention of the two drugs may aggravate the immunosuppression and increase the chance of infection in the later stage. However, there were contrary opinions that sirolimus-based GVHD prophylaxis significantly reduced CMV reactivation (26). Additionally, SRL also exerts anti-EB viral (27) and antifungal (28) actions. Our meta-analysis showed that the SRL-containing regimen neither increased nor decreased bacterial, fungal, and CMV infection. Therefore, more RCTs are needed to confirm this conclusion.

In the seven articles, almost all recipients received identical transplants, including HLA-matched sibling or unrelated donors, with no more than a single allele disparity. Only one paper mentioned mismatched unrelated donor (MMUD); in this report, in the SRL/TAC group, 15% of the patients received MMUD, and in the TAC/MTX group, 22% patients received MMUD (13). Although donor source mismatch unrelated and matched sibling have no difference in OS rate according to the GVHD prophylaxis group, it is worth noting that SRL/TAC has been associated with improved OS among patients at high risk for GVHD based on subgroup analysis (13). There are many factors affecting the clinical outcomes of allo-HSCT, including disease status, conditioning regimen, and GVHD prophylaxis. Whether SRL-based prophylaxis is suitable for haploid transplantation requires additional clinical studies.

Due to different classifications of HSCT, such as HLA-matched or mismatched, sibling, or unrelated donors, the prophylaxis schemes of GVHD are different. To minimize heterogeneity, we strictly chose seven papers for this meta-analysis; from the results, addition of sirolimus could be an effective and safe prophylaxis option for GVHD, although the association of SRL with increased thrombotic complications must be carefully monitored and effectively managed. While SRL has some advantages, it is not a first choice for GVHD prophylaxis; however, subgroup analysis may identify additional advantages in high-risk (≥HCT-CI) groups. More adequately powered RCTs are required to better understand the impact of SRL-based GVHD prophylactic regimens.

## Data Availability Statement

The raw data supporting the conclusions of this article will be made available by the authors, without undue reservation.

## Author Contributions

YF wrote the original draft and revised the manuscript. XC and YF completed the literature search, data extraction, data analysis, and chart making. KC, HS, LW, TC, HY, SY, and XZ edited this review. XC, XZ, and YF funded the work. All authors contributed to the article and approved the submitted version.

## Funding

This work was supported by Chongqing Science and Technology Commission joint project (2021MSXM299) and the Natural Science Foundation of Chongqing (cstc2019jcyj-msxmX0273, cstc2020jcyj-msxmX1086 and cstc2020jcyj-msxmX0448), Science and Technology Innovation Capacity Promotion Project of Army Medical University (2019XLC3014), Special Projects in the Frontier of Military Medicine Natural Science of Xinqiao Hospital (2018 YQYLY002), and National Key Research Program (2017YFA0105502).

## Conflict of Interest

The authors declare that the research was conducted in the absence of any commercial or financial relationships that could be construed as a potential conflict of interest.

## Publisher’s Note

All claims expressed in this article are solely those of the authors and do not necessarily represent those of their affiliated organizations, or those of the publisher, the editors and the reviewers. Any product that may be evaluated in this article, or claim that may be made by its manufacturer, is not guaranteed or endorsed by the publisher.
