# Supplementary material for: The Addition of Sirolimus to GVHD Prophylaxis After Allogeneic Hematopoietic Stem Cell Transplantation: A Meta-Analysis of Efficacy and Safety
Source: Front Oncol. 2021 Sep 9;11:683263. doi: 10.3389/fonc.2021.683263 (PMC8458935; doi:10.3389/fonc.2021.683263)
Supplement: Supplementary file 1 [file DataSheet_1.docx]

| 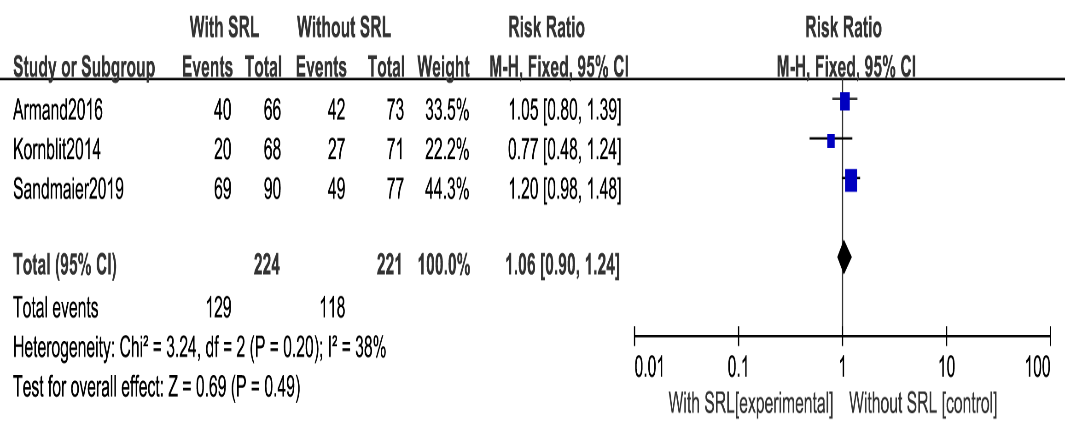  PFS | 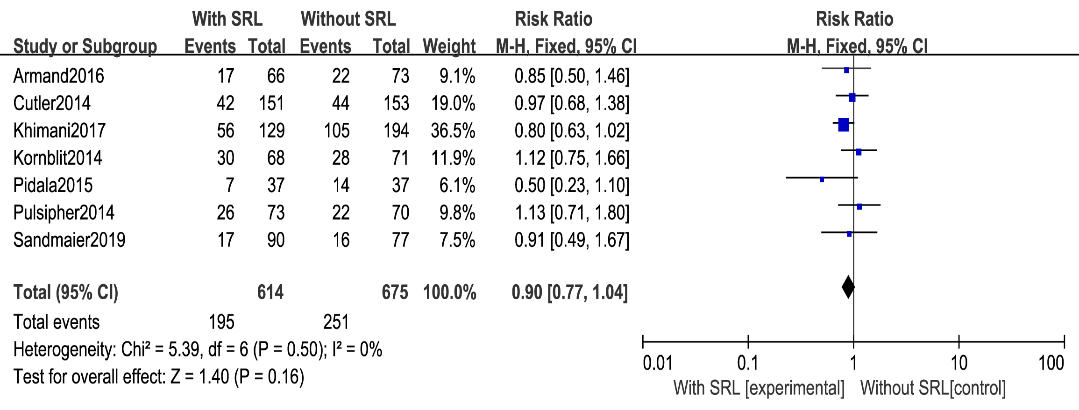  Relapse |
| --- | --- |
| 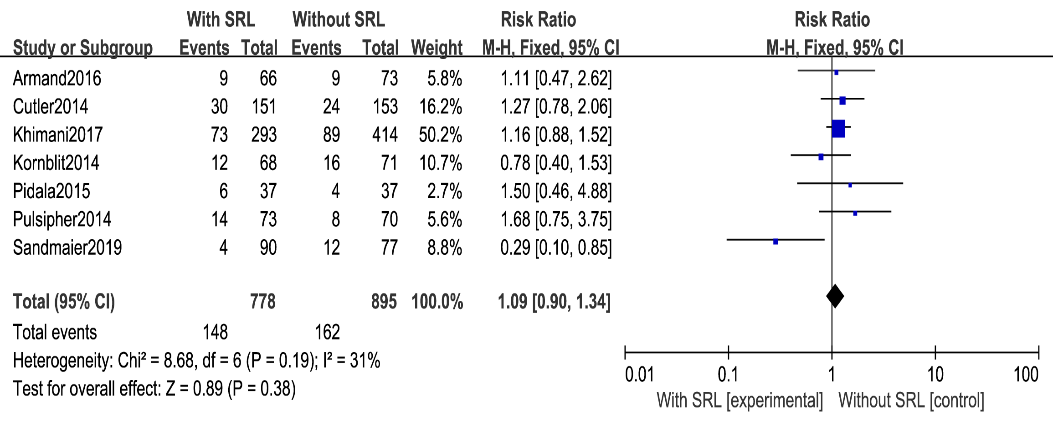  NRM | 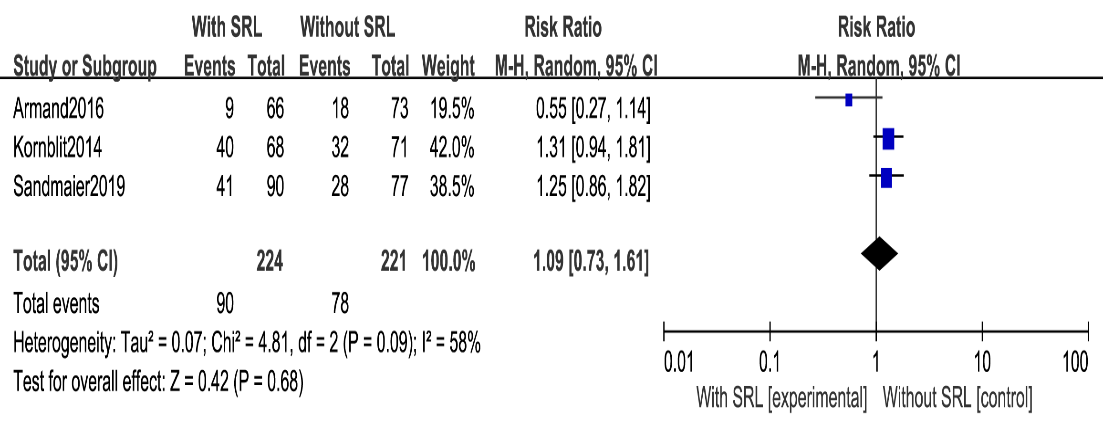  Bacterial infection |
| 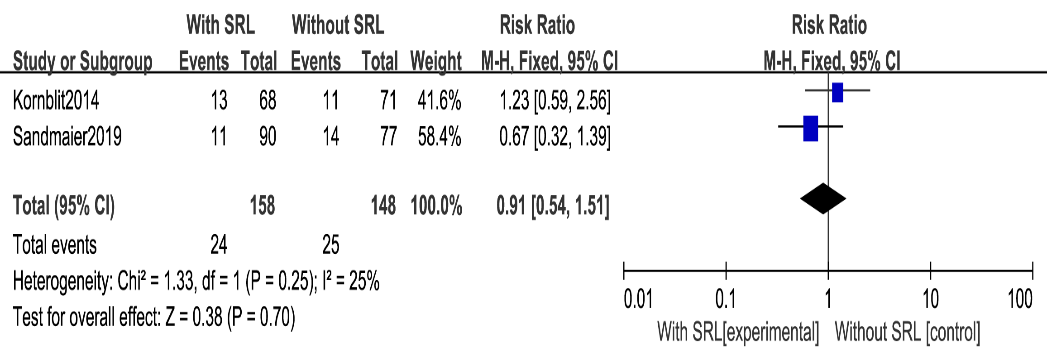  Fungal infection | 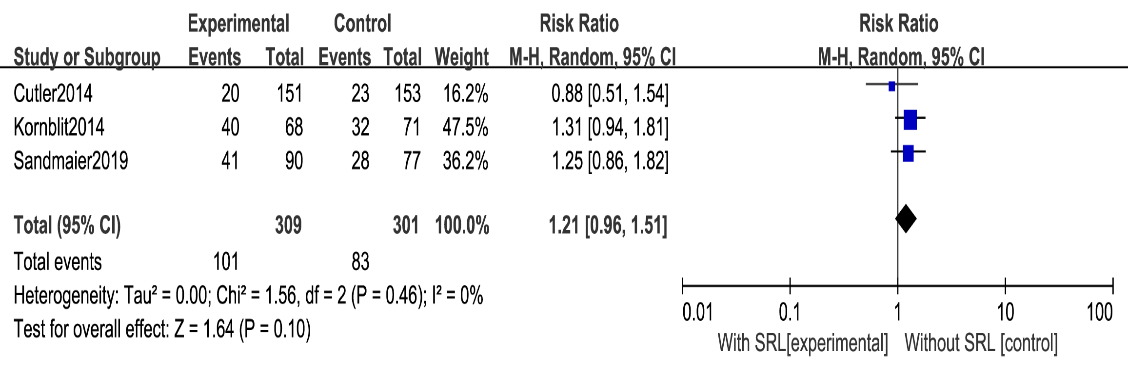  CMV reactivation |

Three studies reported PFS, and the meta result showed there is no differences in PFS (RR, 1.06; 95% CI, 0.90-1.24, *P*=0.49). SRL intervention had no statistical effect on Relapse(*P*=0.16) and NRM(*P*=0.38) . An analysis of the pooled data revealed that SRL-based prophylaxis did not cause a significant increase in bacterial (*P*=0.68) and fungal (*P*=0.70) infection, and CMV reactivation(*P*=0.10)**.**
