# Supplementary material for: The Addition of Sirolimus to GVHD Prophylaxis After Allogeneic Hematopoietic Stem Cell Transplantation: A Meta-Analysis of Efficacy and Safety
Source: Front Oncol. 2021 Sep 9;11:683263. doi: 10.3389/fonc.2021.683263 (PMC8458935; doi:10.3389/fonc.2021.683263)
Supplement: Supplementary file 2 [file DataSheet_2.docx]

**Publication bias assessment**:


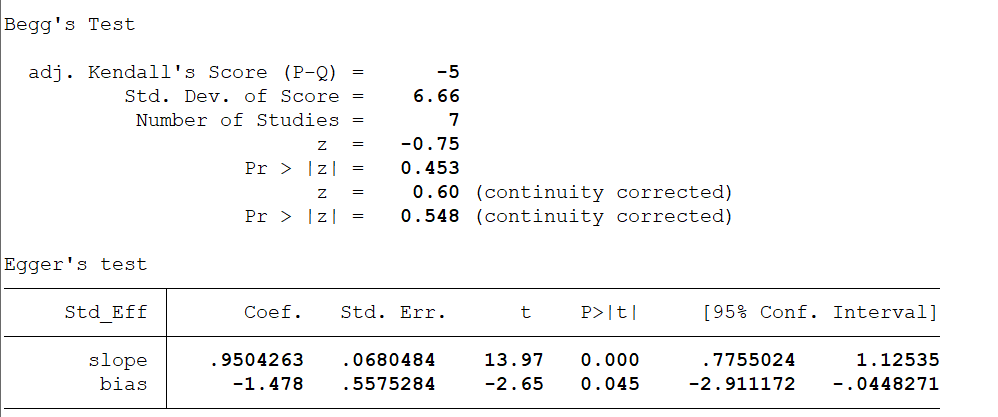


From the above analysis, there was no significant publication bias about II-IV aGVHD（*P*=0.548＞0.05）. If the P > 0.05, there is no obvious publication bias. If the P < 0.05, there is obvious publication bias. We have done the same analysis for other indicators, the results showed that there was no significant publication bias.

| 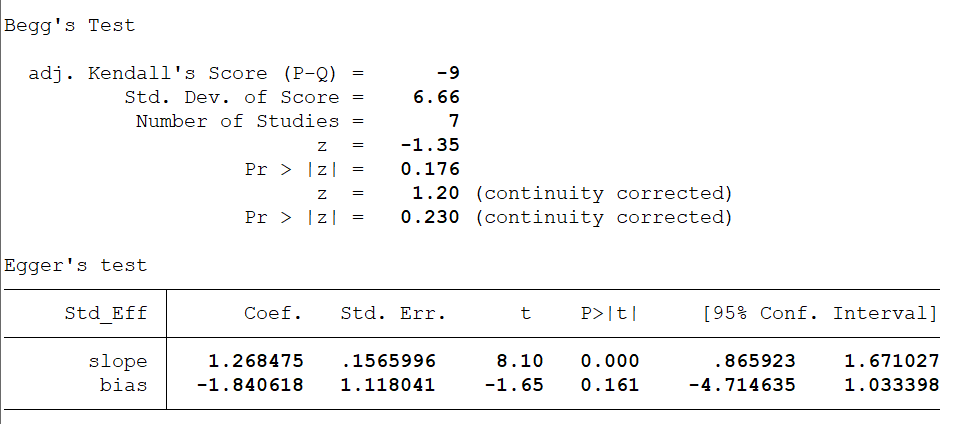  cGVHD | 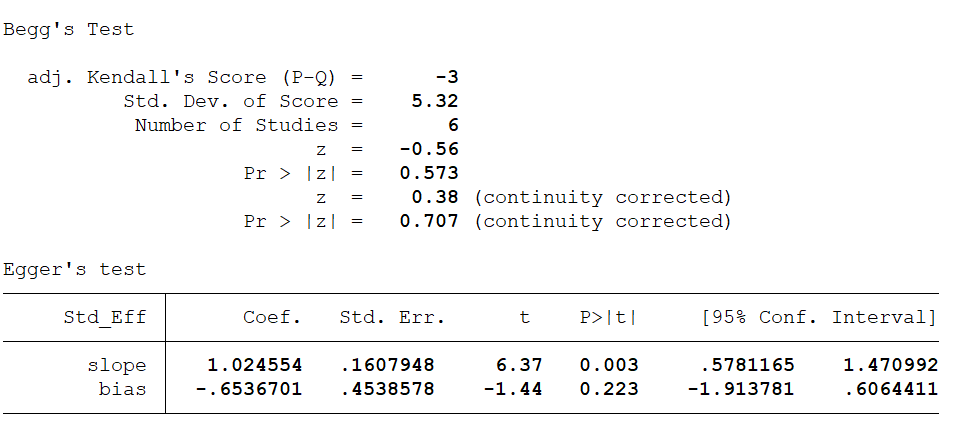  III-IV aGVHD |
| --- | --- |
| 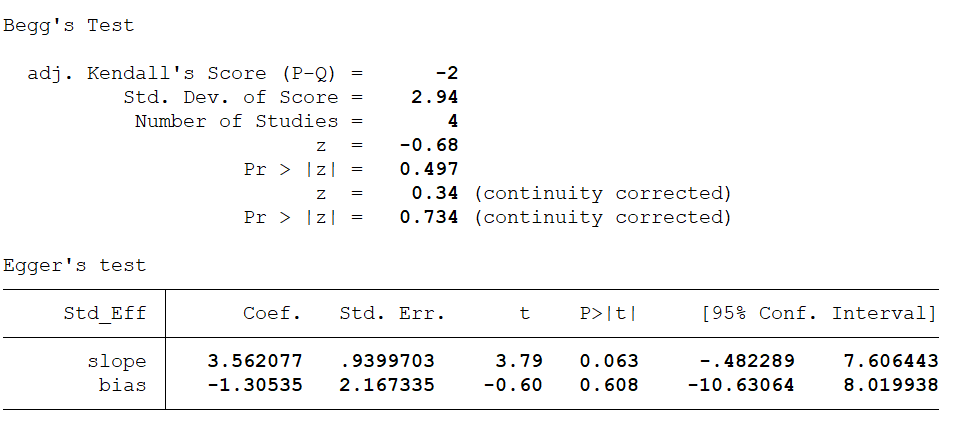  VOD | 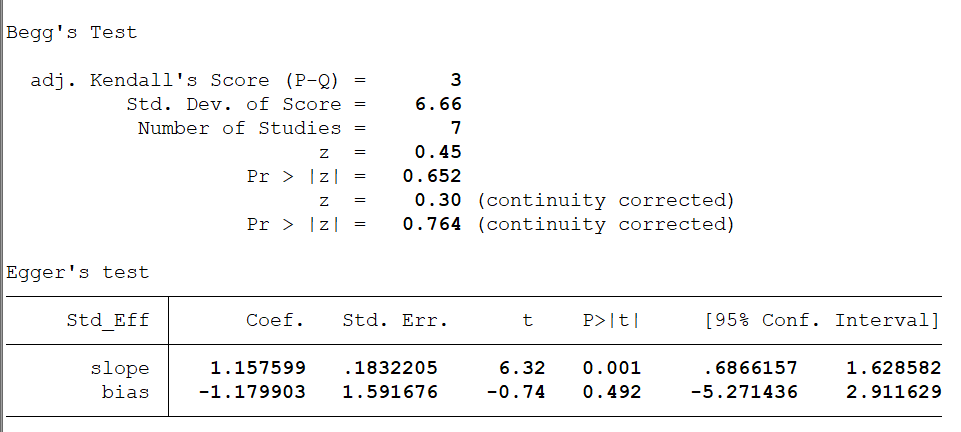  OS |

**Sensitivity analysis:**

We used sensitivity analysis with Stata software to determine the stability of the results. The sensitivity analysis showed the research results were stable and reliable

| 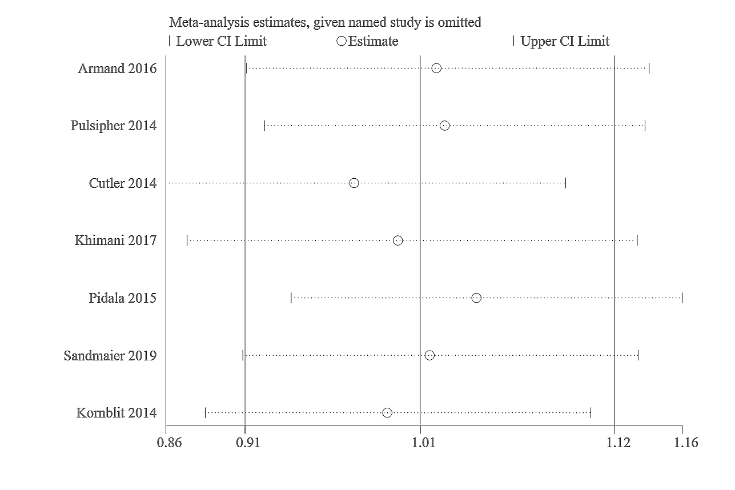  cGVHD | 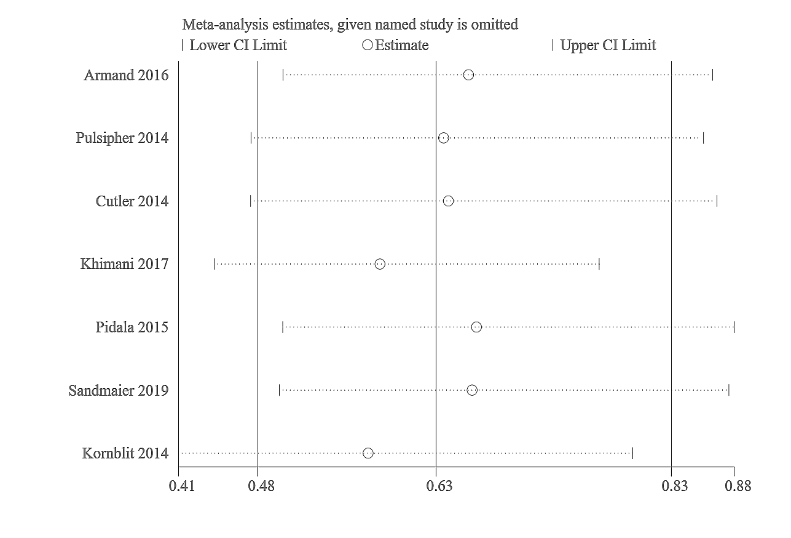  II -IV aGVHD |
| --- | --- |
